# Supplementary material for: Trends and variability in the Southern Annular Mode over the Common Era
Source: Nat Commun. 2023 Apr 22;14:2324. doi: 10.1038/s41467-023-37643-1 (PMC10122664; doi:10.1038/s41467-023-37643-1)
Supplement: Supplementary file 1 — Supplementary Information [file 41467_2023_37643_MOESM1_ESM.pdf]

Supplement to “Trends and variability in the Southern Annular Mode over the Common Era”

Jonathan King[1,2*], Kevin J. Anchukaitis[1,2,3], Kathryn Allen[4,5,6], Tessa Vance[7] and Amy Hessl[8]

[1]Department of Geosciences, University of Arizona, Tucson, AZ 85721 USA.

[2]Laboratory of Tree-Ring Research, University of Arizona, Tucson, AZ 85721 USA.

[3]School of Geography, Development, and Environment, University of Arizona, Tucson, AZ 85721 USA.

[4]School of Geography, Planning and Spatial Sciences, University of Tasmania, Hobart, Australia 7001.

[5]School of Ecosystem and Forest Sciences, University of Melbourne, Richmond, VIC Australia 3121.

[6]Centre of Excellence for Australian Biodiversity and Heritage, University of New South Wales, Australia.

[7]Australian Antarctic Program Partnership, Institute for Marine and Antarctic Studies, University of Tasmania, Hobart, Australia.

[8]Department of Geology and Geography, West Virginia University, Morgantown, WV USA.

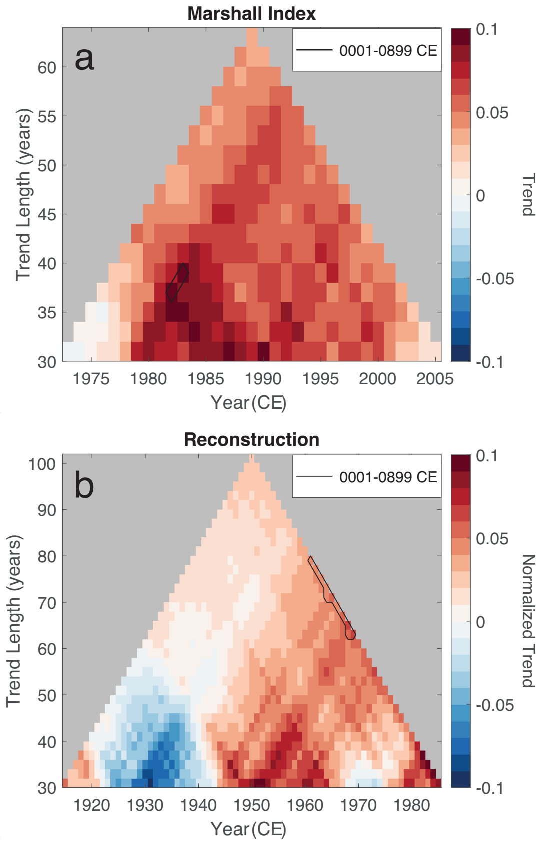

Supplemental Figure 1: Trend analysis using 1-899 CE as the statistical background. (a) Instrumental trends for the Marshall index. Colored squares indicate trend values calculated over different periods. Values are calculated from a window centered on the year denoted on the X axis. The length of trend is given by the duration on the Y axis. Solid contours surround trends that are significantly different (at the 95% confidence level) from the natural distribution of trends in the reconstruction over the period 1-899 CE. (b) As in panel a, but for trends calculated from the reconstruction. The units of the reconstruction (and thereby its trends) have been normalized to the Marshall index, such that mean and variance of the detrended reconstruction match those of the detrended Marshall index over the interval 1958-2000 CE.

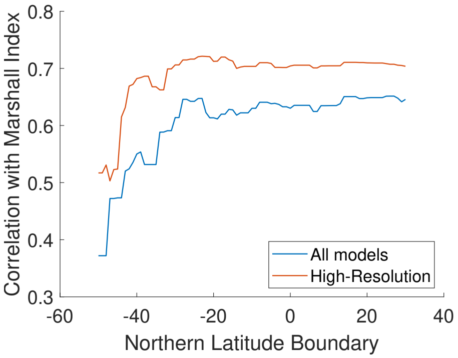

Supplemental Figure 2: Correlation of reconstructions with the Marshall Index for different latitude cutoff boundaries. Correlation is assessed over the period 1958-2000 CE. Blue line shows results for reconstructions with a prior constructed from all 10 models. Red line shows results for reconstructions with a prior built from the 4 high-resolution models.

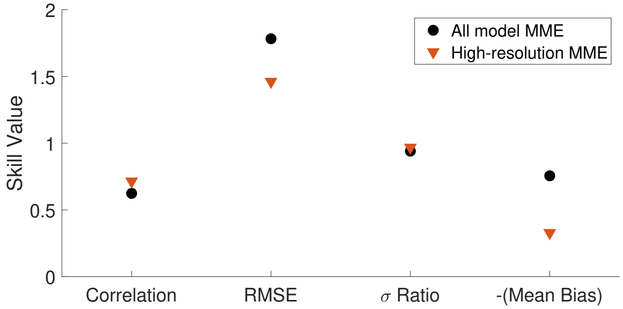

Supplemental Figure 3: Skill comparison for different priors. Skill metrics are assessed relative to the Marshall index over the period 1958-2000. Prior to skill assessment, reconstruction time series are scaled such that the mean and standard deviation of the detrended reconstructions match the mean and standard deviation of the detrended Marshall index. Black circles indicate values for the all-model prior (10 models). Red triangles indicate values for the high-resolution multi-model prior (4 models). Figure columns show (1) Correlation, (2) Root mean-square error, (3) Standard deviation ratios (computed as the ratio of reconstruction variability over Marshall index variability), and (4) Bias in the mean value of the series. To condense the plot, negative mean biases are shown here.

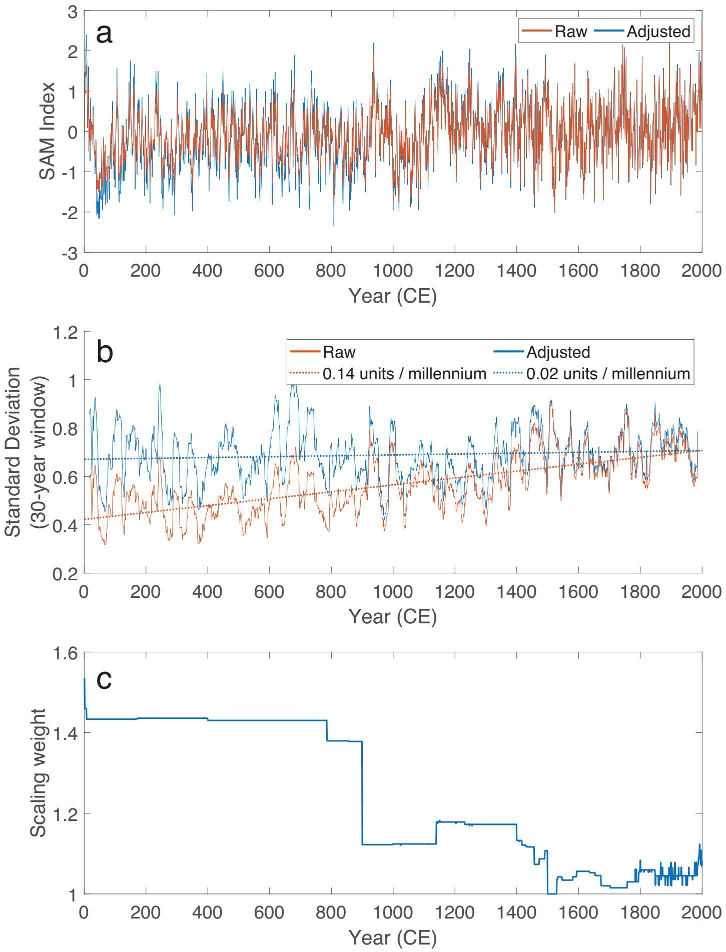

Supplemental Figure 4: Variance correction scheme. (a) Raw assimilation (red) and the variance-adjusted final reconstruction (blue). The raw assimilation displays less variance than the final reconstruction, particularly during the early part of the record. (b) Solid lines show the moving 30-year standard deviations of the raw assimilation (red) and variance-adjusted reconstruction (blue). Dotted lines indicate the linear trends in the moving standard deviation time series over the full period. (c) The multiplicative scaling weights used to produce the final, variance-adjusted reconstruction. Weights are applied multiplicatively to the raw assimilation in each time step.

	Climate Model	Acronym	Output Resolution (Latitude x Longitude)	Experiments	Number of Years
High-Resolution	CCSM4	CCSM4	0.94° x 1.25°	past1000, historical	1156
	CESM1.1-CAM5	CESM	1.89° x 2.50°	LME full-forcing 2	1156
	MPI-ESM-P	MPI	1.86° x 1.88 °	past1000, historical	1156
	MRI-CGCM3	MRI	1.12° x 1.13°	past1000, historical	1156
	BCC-CSM1-1	BCC	2.79° x 2.81°	past1000	1151
	CSIRO-Mk3L-1-2	CSIRO	3.18° x 5.63°	past1000, historical	1150
	FGOALS-gl	FGOALS	4.62° x 5.00°	past1000	1000
	HadCM3	HadCM3	2.50° x 3.75°	past1000, historical	1147
	IPSL-CM5A-LR	IPSL	1.89° x 3.75°	past1000, historical	1156
	MIROC-ESM	MIROC	2.79° x 2.81°	past1000, historical	1156

Supplemental Table 1: Climate models tested for use in assimilation priors.

Model	SAM Latitudes	Correlation
BCC	43°S, 66°S	-0.90
CCSM4	47°S, 69°S	-0.79
CESM	46°S, 69°S	-0.89
CSIRO	43°S, 65°S	-0.90
HadCM3	45°S, 68°S	-0.84
FGOALS	31°S, 59°S	-0.84
IPSL	39°S, 63°S	-0.94
MIROC	40°S, 63°S	-0.90
MPI	42°S, 66°S	-0.83
MRI	44°S, 69°S	-0.90
Gong et al. (1999)	40°S, 65°S	

Supplemental Table 2: Latitudes with the most strongly anticorrelated zonal-mean sea level pressure anomalies in tested climate models. Correlation coefficients are calculated for austral summer (December - February) seasonal means over all available years in the interval 850-2005 CE.

Proxy Type	PAGES2k ID	Site Name	Latitude, Longitude	Years (CE)	Season
Coral	Ocn 114	Houtman Abrolhos	28.47°S, 113.77°E	1795 - 1994	July - June
	Ocn 153	Houtman Abrolhos	28.46°S, 113.77°E	1798 - 2000	January - December
	Ocn 154	Houtman Abrolhos	28.46°S, 113.75°E	1848 - 2000	January - December
	Ocn 155	Houtman Abrolhos	28.46°S, 113.75°E	1848 - 2000	January - December
	Ocn 158	Houtman Abrolhos Islands	28.47°S, 113.77°E	1900 - 2000	January - December
Glacier Ice	Ant 001	Talos Dome	72.80°S, 159.06°E	1232 - 1995	January - December
	Ant 002	DSS	66.77°S, 112.81°E	173 - 1995	January - December
	Ant 003	Plateau Remote	84.00°S, 43.00°E	2 - 1986	January - December
	Ant 004	Coastal DML	70.86°S, 11.54°E	1533 - 1994	January - December
	Ant 005	Site DML05	75.00°S, 0.01°W	166 - 1996	January - December
	Ant 006	WDC05A	79.46°S, 112.09°W	786 - 2000	January - December
	Ant 007	WDC06A	79.46°S, 112.09°W	1 - 2000	July - June
	Ant 008	US-ITASE-2000-1	79.38°S, 111.24°W	1673 - 2000	July - June
	Ant 010	James Ross Island	64.20°S, 57.68°W	1 - 2000	January - December
	Ant 011	Siple Station	75.92°S, 84.25°W	1417 - 1983	January - December
	Ant 012	Berkner Island (South)	79.57°S, 45.72°W	1000 - 1992	January - December
	Ant 017	Ferrigno	74.57°S, 86.90°W	1703 - 2000	January - December
	Ant 019	MES	77.52°S, 167.68°E	1473 - 2000	January - December
	Ant 020	Site DML07	75.58°S, 3.43°W	1000 - 1994	January - December
	Ant 021	Site DML17	75.17°S, 6.50°E	1000 - 1997	January - December
	Ant 024	US-ITASE-2002-4	86.50°S, 107.99°W	1594 - 2000	July - June
	Ant 025	VLG	77.33°S, 162.53°E	1140 - 2000	January - December
	Ant 026	Vostok	78.28°S, 104.80°E	1654 - 2000	January - December
	Ant 028	WDC06A	79.46°S, 112.09°W	1 - 2000	July - June
Borehole	Ant 027	WAIS-Divide	79.46°S, 112.12°W	8 - 2000	January - December

Continued on next page

Continued from previous page

Proxy Type	PAGES2k ID	Site Name	Latitude, Longitude	Years (CE)	Season
Lake Sediment	SAm 003	Laguna Aculeo	33.85°S, 70.92°W	856 - 1997	December - February
	SAm 030	Laguna Chepical	32.27°S, 70.50°W	1 - 2000	November - February
	SAm 031	Laguna Escondida	45.52°S, 71.82°W	400 - 2000	January - December
Tree	Aus 001	Mt. Read	41.83°S, 145.53°E	1 - 2000	November - April
	Aus 002	Oroko	43.23°S, 170.28°E	900 - 1999	September - April
	Aus 004	CTP East Tasmania	41.31°S, 147.75°E	1430 - 1994	September - November
	Aus 005	Pink Pine NZ	43.00°S, 171.00°E	1457 - 1999	September - April
	Aus 007	Buckleys Chance Tasmania	42.27°S, 145.87°E	1463 - 1991	October - April
	Aus 009	CTP West Tasmania	41.67°S, 145.65°E	1547 - 1998	June - August
	Aus 030	Stewart Island	47.00°S, 167.80°E	1758 - 1993	September - April
	Aus 031	Takapari Cedar	40.07°S, 175.98°E	1530 - 1992	September - April
	SAm 006	Central Andes composite 11	40.10°S, 72.05°W	1492 - 1995	May - April
	SAm 024	Central Andes composite 6	38.50°S, 71.50°W	1435 - 2000	May - April
	SAm 025	Central Andes composite 9	39.33°S, 71.25°W	1636 - 2000	May - April
	SAm 029	Central Andes composite 15	41.17°S, 71.92°W	1582 - 1991	May - April

Supplemental Table 3: PAGES2k Sites used in the final reconstruction. The included sites are south of 25°S, and have annual or higher resolution.
